# A Hollow-Structured Manganese Oxide Cathode for Stable Zn-MnO_2_ Batteries

**DOI:** 10.3390/nano8050301

**Published:** 2018-05-05

**Authors:** Xiaotong Guo, Jianming Li, Xu Jin, Yehu Han, Yue Lin, Zhanwu Lei, Shiyang Wang, Lianjie Qin, Shuhong Jiao, Ruiguo Cao

**Affiliations:** 1 School of Environmental and Materials Engineering, Yantai University, Yantai 264005, China; aguoxiaotong@hotmail.com (X.G.); ahanyehu@hotmail.com (Y.H.); 2 Research Institute of Petroleum Exploration and Development (RIPED), PetroChina, Beijing 100083, China; jinxu@petrochina.com.cn; 3 Hefei National Laboratory for physical Science at the Microscale, University of Science and Technology of China, Hefei 230026, China; linyue@ustc.edu.cn; 4 Key Laboratory of Materials for Energy Conversion Chinese Academy of Sciences (CAS), Department of Materials Science and Engineering, University of Science and Technology of China, Hefei 230026, China; zwlei@mail.ustc.edu.cn (Z.L.); wangshiy@ustc.edu.cn (S.W.); jiaosh@pku.edu.cn (S.J.)

**Keywords:** manganese oxide, hollow structure, multivalent intercalation, zinc ion batteries

## Abstract

Aqueous rechargeable zinc-manganese dioxide (Zn-MnO_2_) batteries are considered as one of the most promising energy storage devices for large scale-energy storage systems due to their low cost, high safety, and environmental friendliness. However, only a few cathode materials have been demonstrated to achieve stable cycling for aqueous rechargeable Zn-MnO_2_ batteries. Here, we report a new material consisting of hollow MnO_2_ nanospheres, which can be used for aqueous Zn-MnO_2_ batteries. The hollow MnO_2_ nanospheres can achieve high specific capacity up to ~405 mAh g^−1^ at 0.5 C. More importantly, the hollow structure of birnessite-type MnO_2_ enables long-term cycling stability for the aqueous Zn-MnO_2_ batteries. The excellent performance of the hollow MnO_2_ nanospheres should be due to their unique structural properties that enable the easy intercalation of zinc ions.

## 1. Introduction

Lithium-ion batteries (LIBs) have predominantly held a significant share of the energy storage market for portable electronics and electric vehicles since the 1990s, due to their high energy/power density and long cycling life. However, with the rapid development of renewable energy plants, there is an extensive and urgent demand for energy storage technologies for large-scale smart grid applications, which require rechargeable battery systems with good cycling performance, low cost, high safety, and environmental friendliness. In searching for new chemistry beyond lithium-ion batteries, multivalent secondary batteries (Mg, Ca, Zn, and Al) have attracted tremendous research efforts, which could, in principle, deliver a higher energy density based on their multi-electron reaction mechanisms [1,2]. Among the multivalent batteries based on intercalation chemistries, aqueous rechargeable zinc ion batteries are considered as a promising candidate for large-scale energy storage applications because of their low cost and the large abundance of Zn [3]. In addition, the aqueous electrolytes in zinc ion batteries provide better safety compared to other battery systems with flammable organic electrolytes. However, the development of aqueous zinc ion batteries is significantly hindered by the limited choice of positive electrode materials, which usually suffer from low specific capacity and poor cycling stability [4]. Many failure mechanisms are associated with phase transformations and the formation of irreversible products [5,6]. Only a few positive electrodes coupled with suitable electrolytes have been demonstrated to be able to achieve stable long-term cycling for aqueous zinc ion batteries [7,8,9,10,11,12].

Despite their low cost and high abundance, manganese oxides have a variety of advantages including tunable crystal structure and a scalable manufacturing process, which have been widely used for many energy storage applications including lithium-ion batteries, supercapacitors, and zinc-air batteries [13,14,15]. Manganese oxides possess a variety of polymorphs, including α-, β-, γ-, δ-, λ-, and ε-types, which form different structures such as tunnel, layered, and spinel structures, and can be used as positive electrode materials for aqueous zinc manganese dioxide (Zn-MnO_2_) batteries [16,17,18,19]. Birnessite-type manganese dioxide (δ-MnO_2_) is featured with a layered structure, which is considered as a favorable host for the intercalation of various cations [20,21]. Considerable efforts have been made to verify this layered structure materials for reversible zinc ion intercalation [22]. It was observed that the birnessite-type manganese dioxide is not stable as a positive electrode material under the long-term cycling of a secondary Zn-MnO_2_ battery [23]. In order to deliver a two-electron capacity for a long cycling life, the structure of δ-MnO_2_ needs to be maintained by structure-stabilizing agents. For example, it was reported that the birnessite-type MnO_2_ could achieve a full two-electron capacity for over 6000 cycles when mixed with bismuth oxide (Bi_2_O_3_), called Bi-birnessite (Bi-δ-MnO_2_), intercalated with Cu^2+^ ions [24]. Also, we note that hollow nanostructures offer promising potentials for energy storage applications because of their favorable properties in terms of hierarchical structure complexity and fast ion transport pathway [25,26].

Herein, without stabilizing agents, we tackle the stability issue of δ-MnO_2_ in aqueous Zn-MnO_2_ batteries by tuning the nanostructure of this materials. A hollow spherical structure of δ-MnO_2_ is developed to enable a robust architecture and a high specific capacity of the positive electrode for an aqueous Zn-MnO_2_ battery. The hollow manganese oxide cathode exhibits high capacity and stable cycling performance with an aqueous electrolyte.

## 2. Materials and Methods

### 2.1. Synthesis of Hollow Spherical MnO_2_ Particles

SiO_2_ spherical particles were prepared by a sol-gel method and used as a template. In a typical synthesis procedure, 4.0 mL of tetrapropyl orthosilicate was added into the mixture of ethanol (50.0 mL), water (10.0 mL), and ammonia (1.0 mL, 25–28%) at room temperature under stirring. After 14 h, the obtained SiO_2_ suspension was centrifuged, rinsed with distilled water, and re-dispersed in 30 mL H_2_O to form a SiO_2_ white suspension.

Then, 0.98 g of KMnO_4_ was added to the SiO_2_ suspension and followed by ultrasonic treatment for 30 min. The suspension was then transferred to a Teflon-lined autoclave and heated at 150 °C for 48 h. The brown product with a silica/manganese oxide core-shell structure (SiO_2_@MnO_2_) was obtained and then etched in the 2.00 M of NaCO_3_ solution at 60 °C for 24 h.

After the removal of the SiO_2_ core, the final products of the hollow spherical MnO_2_ particles were collected by centrifugation, washed with deionized water, and freeze-dried.

### 2.2. Cell Assembly and Test

To prepare the cathode electrode, the slurry was prepared with 70 wt % MnO_2_, 20 wt % KB (Ketjenblack), and 10 wt % PVDF (Polyvinylidene Fluoride) binder and casted onto a Ti foil current collector. The electrode was dried at 60 °C in a vacuum oven for 24 h. The loading of MnO_2_ on the electrodes was around 0.5 mg/cm^2^. The CR2032 coin cells were assembled with zinc metal as anodes and MnO_2_ as cathodes. The electrolyte was 1.0 M ZnSO_4_ with 0.2 M MnSO_4_ as an additive and glass fiber was used as the separator. Galvanostatic measurements were carried out between 1.0 and 1.8 V on a Land CT2001A system (LANHE, Wuhan, China). The cyclic voltammetry (CV) experiments were performed with a CHI600E electrochemical workstation (CH, Shanghai, China) at a scanning rate of 0.1 mV s^−1^ between 0.8 and 1.9 V. The electrochemical impedances spectroscopy (EIS) of the active material was recorded on an electrochemical workstation (Solartron) using the frequency response analysis with a range from 100 kHz to 0.01 Hz.

### 2.3. Materials Characterization

The dimensions and morphologies were examined using scanning electron microscopy (SEM, JSM-2100F, JEOL, Tokyo, Japan. The crystallographic structures were investigated by powder XRD (X-ray diffraction) measurements on a Rigaku D/max-TTR III diffractometer with Cu Kα radiation (Rigaku Corporation, Shibuya-ku, Japan), 40 kV, 200 mA. The nanostructures of hollow spherical samples were characterized by high-resolution transmission electron microscopy (HRTEM, JEOL, Tokyo, Japan, 2010).

## 3. Results

**Figure 1:**
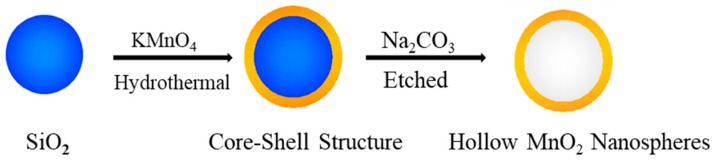
Schematic illustration of the synthetic process of hollow MnO_2_ nanospheres.

The hollow MnO_2_ nanospheres were synthesized using a template approach. The synthesis process of hollow MnO_2_ nanospheres is illustrated schematically in Figure 1. First, the SiO_2_ nanospheres were prepared through a sol-gel method. To form the core-shell structure of SiO_2_@MnO_2_, the as-synthesized SiO_2_ nanospheres were used as templates for a hydrothermal process with a KMnO_4_ solution. After being etched in an aqueous Na_2_CO_3_ solution, the SiO_2_ core was removed and the hollow MnO_2_ nanospheres were obtained for characterization and electrochemical tests.

**Figure 2:**
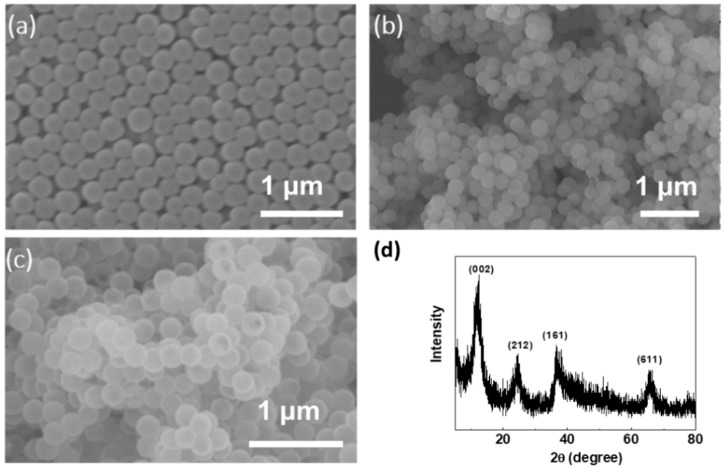
SEM images of SiO_2_ nanospheres (**a**); SiO_2_@MnO_2_ core-shell structure (**b**); and hollow MnO_2_ nanospheres (**c**); (**d**) XRD patterns of the hollow MnO_2_ nanospheres.

As shown in Figure 2a, the prepared monodisperse SiO_2_ nanospheres show a uniform sphere morphology with a size ranging from 200 to 250 nm. After the reaction with aqueous KMnO_4_ solution by a hydrothermal process at 150 °C for 48 h, the core-shell structure of SiO_2_@MnO_2_ was formed (Figure 2b). It was clearly shown that the SiO_2_ nanospheres were fully covered with MnO_2_ and no aggregation was observed. The uniform coating on SiO_2_ nanoparticles was due to the surface-induced nucleation and growth of manganese oxide species. To remove the SiO_2_ core materials, the core-shell SiO_2_@MnO_2_ particles were etched in an aqueous 2 M Na_2_CO_3_ solution for 24 h. After the etching process, very little silica is remained based on EDX (Energy Dispersive X-Ray Spectroscopy) and XPS (X-ray Photoelectron Spectroscopy) measurements. Figure 2c shows the typical morphology of hollow spherical MnO_2_ particles after the etching treatment. It is clearly seen that the spherical morphology is completely maintained and almost no damage was observed on the shell structure of MnO_2_. Powder X-ray diffraction (XRD) measurement was used to examine the crystallographic structure phase in the as-synthesized hollow MnO_2_ spheres. Figure 2d shows the XRD pattern of the as-synthesized hollow MnO_2_ nanospheres, which shows peaks at 2θ around 12.4°, 24.8°, 36.8°, and 65.8°. These peaks can be indexed to birnessite-type MnO_2_. The peaks lack the long-range order of layers and a tail toward higher angle two-theta, demonstrating common features of the birnessite structure [27].

**Figure 3:**
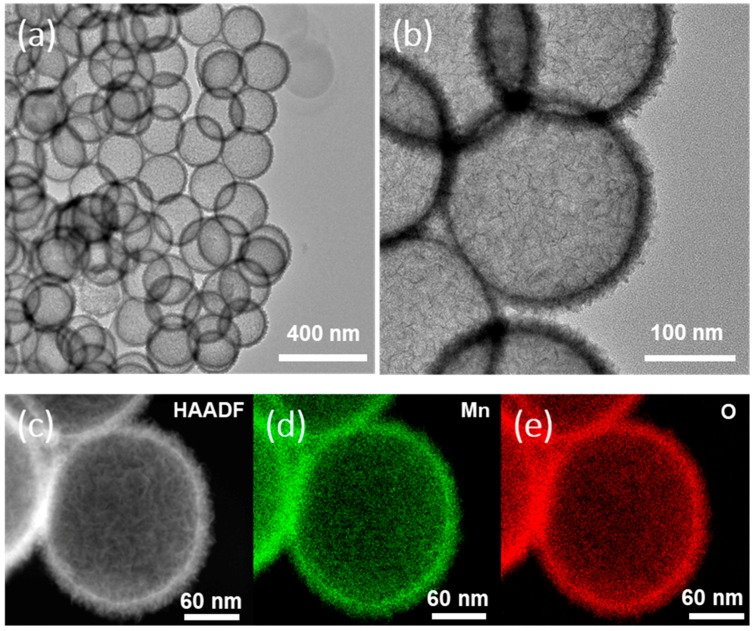
High- (**a**) and low-magnification (**b**) HRTEM images of hollow MnO_2_ nanospheres; (**c**) HAADF-STEM image of hollow MnO_2_ nanospheres. Elemental mapping of hollow MnO_2_ nanospheres: (**d**) Mn and (**e**) O.

**Figure 4:**
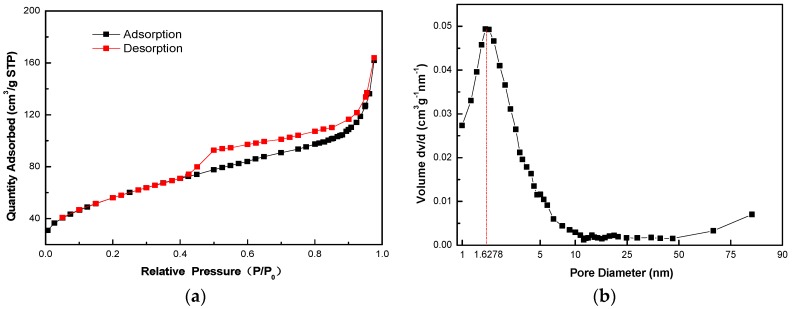
BET measurement of hollow MnO_2_ nanospheres. (**a**) Nitrogen adsorption/desorption isotherms of as-synthesized hollow MnO_2_ nanospheres; (**b**) the pore size distribution of hollow MnO_2_ nanospheres, as calculated using a BJH (Barrett-Joyner-Halenda) method.

In order to further investigate the structure of the as-synthesized hollow MnO_2_ nanospheres, we carried out high-resolution TEM analysis. Figure 3a clearly shows the hollow structure of MnO_2_ nanospheres without aggregation observed. The MnO_2_ shell is around 15 nm thick and its diameter is around 200 nm. Almost no damage was observed under TEM analysis, indicating that the shell structure is robust enough to tolerate the harsh etching process. Detailed analysis shows that the shell structure consists of very thin nanosheets of MnO_2_, which form interconnected wrinkle structures (Figure 3b). The wrinkled structure was confirmed by HAADF-STEM (High-Angle Annular Dark Field Scanning Transmission Electron Microscopy) image (Figure 3c). Moreover, elemental compositions of the hollow MnO_2_ structure were mapped by electron energy loss spectroscopy (EELS), confirming the uniform dispersion of elemental Mn and O (Figure 3d,e). A N_2_ adsorption/desorption analysis of hollow MnO_2_ nanospheres was conducted to analyze the surface area of the wrinkled hollow structure. The BET (Brunauer-Emmett-Teller) surface area of as-synthesized hollow MnO_2_ nanosphere was ~200 m^2^/g with a pore size distribution at ~1.6 nm (Figure 4), indicating that the hollow MnO_2_ nanosphere also featured a microporous structure.

**Figure 5:**
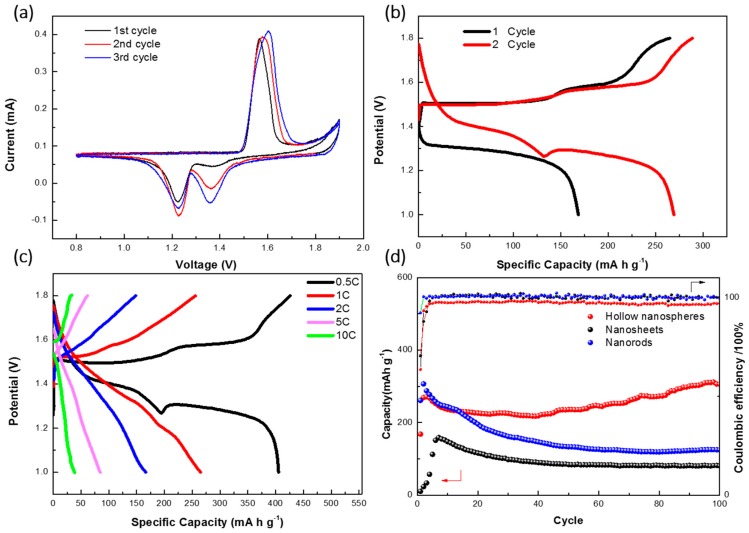
Electrochemical performance of Zn-MnO_2_ batteries: (**a**) CV profiles; (**b**) typical charge–discharge curves; (**c**) rate performance; and (**d**) long-term cycling stability of hollow MnO_2_ nanospheres, MnO_2_ nanosheets, and MnO_2_ nanorods at 1 C with an electrolyte of 1.0 M Zn(SO_4_)_2_ and 0.2 M MnSO_4_.

The electrochemical performance of hollow MnO_2_ nanospheres was evaluated in aqueous Zn-MnO_2_ batteries. The Zn-MnO_2_ cell was assembled with zinc foil as an anode and 1.0 M Zn(SO_4_)_2_ aqueous solution with 0.2 M MnSO_4_ as an electrolyte. Figure 5a shows the cyclic voltammetry scan results of the Zn-MnO_2_ cell with hollow MnO_2_ nanospheres as cathode materials. The sweep range was between 1.9 V and 0.8 V vs. Zn/Zn^2+^, and the sweep rate was 0.1 mV/s. During the first cycle, a low cathodic peak at around 1.36 V and a sharp cathodic peak at around 1.22 V were observed, while only one anodic peak at around 1.58 V was observed when sweeping back. In the following scan cycles, the cathodic peak at 1.36 V increased gradually, indicating an activation process of hollow MnO_2_ nanospheres during discharge. Figure 5b shows the typical galvanostatic discharge/charge profiles of the Zn-MnO_2_ cell at a 1 C rate. The discharge curve in first cycle exhibited a flat plateau at around 1.26 V, which is consist with the CV results. Two plateaus, ~1.38 V and ~1.26 V, were observed during the second discharge process, which are related to two distinct cathodic peaks in the second sweep of CV curves, indicating a two-step intercalation process of zinc ions into the birnessite structure. Upon charge process, two plateaus at ~1.50 V and ~1.58 V were observed. Previously, the two-step intercalation process was also observed in other Zn-MnO_2_ batteries based on birnessite-type materials [23,24]. The discharge capacity of hollow MnO_2_ nanospheres reached up to ~270 mAh g^−1^ at a 1 C rate.

**Figure 6:**
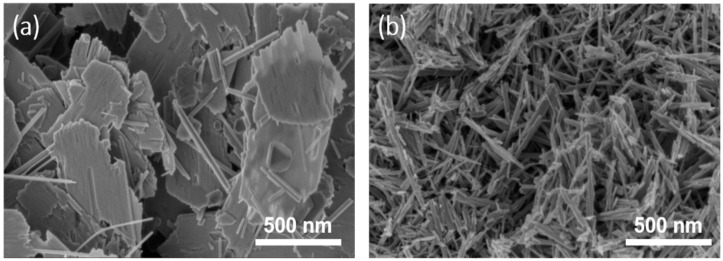
SEM images of (**a**) MnO_2_ nanosheets and (**b**) MnO_2_ nanorods.

Figure 5c shows the typical charge/discharge profiles of Zn-MnO_2_ batteries at different current densities. At rates of 0.5, 1, 2, 5, and 10 C, specific discharge capacities of ~405, ~265, ~166, ~85, and ~40 mAh g^−1^ were obtained, respectively, indicating a good rate performance of the hollow MnO_2_ nanospheres. The long-term cycling performance of the Zn-MnO_2_ batteries in terms of discharge capacity and coulombic efficiency was also investigated at 1 C. As shown in Figure 5d, we compared the cycling performance of nanosheets, nanorods, and hollow spherical structure of MnO_2_ in aqueous Zn-MnO_2_ batteries. The morphologies of MnO_2_ nanosheets and nanorods are shown in Figure 6. The initial discharge capacity for hollow MnO_2_ nanospheres was ~168 mAh g^−1^. After the activation process, the discharge capacity of the second cycle was reached at ~270 mAh g^−1^. Notably, after 100 cycles, the discharge capacity was stabilized at ~305 mAh g^−1^ with a coulombic efficiency over 97%. However, the MnO_2_ nanorods showed a quickly fading capacity. The MnO_2_ nanosheets performed a low discharge capacity and poor cycling performance. The excellent rate capability and cycling stability of the Zn-MnO_2_ cell should be due to the hollow structure of the birnessite-type MnO_2_ cathode materials.

**Figure 7:**
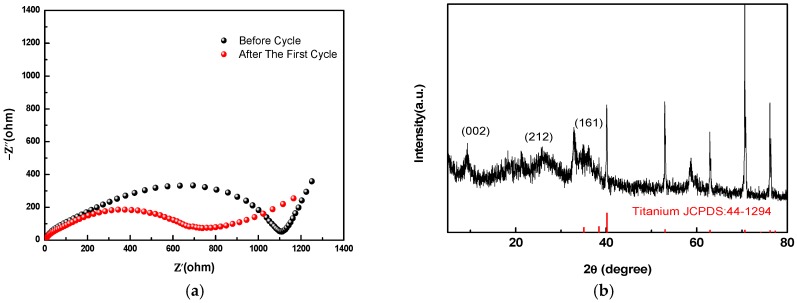
(**a**) Electrochemical impedance spectra of the Zn/MnO_2_ cells before any cycles and after the first cycle; (**b**) XRD patterns of the cathode after the first cycle.

EIS measurements were performed to evaluate the impedance difference between before cycle and after the first discharge/charge cycle. As depicted in Figure 7a, the charge transfer impedance decreased after the first cycle, which indicated that the intercalation of Zn^2+^ ions into the MnO_2_ structure became easier after the structure transformation. An ex situ XRD analysis was conducted for the cathode after the first cycle. As shown in Figure 7b, the representative birnessite structure peaks, (002) and (212), significantly decreased in intensity, especially compared to the mixed indices (161) peak. This selective loss suggests a loss of long-range order in the direction of the layers, perhaps due to a structural transformation to another polymorph with similar building blocks but not layered.

## 4. Conclusions

In summary, hollow MnO_2_ nanospheres were synthesized through a facile hydrothermal approach and used as cathode materials in aqueous Zn-MnO_2_ batteries. The hollow birnessite-type MnO_2_ cathode achieved a relatively high discharge capacity and stable cycling performance with an aqueous electrolyte in a Zn-MnO_2_ battery. The excellent electrochemical performance was ascribed to the unique hollow structure, which favors the intercalation process of zinc ions and enables a stable cycling of the Zn-MnO_2_ battery.
